# Health effects of black and blond tobacco: a comparative analysis on smoking-related diseases with emphasis on bronchitis and COPD

**DOI:** 10.3389/fpubh.2026.1782311

**Published:** 2026-06-03

**Authors:** María Bejarano-Vila, Irene Abad-Perez, Daniel Bautista, Francisco Bosch-Morell, Isabel Martinez-Solis

**Affiliations:** 1 SEPLAN Group, Botanical Garden, University of Valencia, Valencia, Spain; 2 Department of Pharmacy, Faculty of Health Sciences, Universidad Cardenal Herrera-CEU, CEU Universities, Valencia, Spain; 3 Clinical Documentation and Admission Unit (CDAU), Consorcio Hospital General Universitario de Valencia (CHGUV), Valencia, Spain; 4 Hospital Universitario Dr. Peset, Valencia, Spain; 5 Department of Biomedical Sciences, Faculty of Health Sciences, and Biomedical Sciences Institute, Universidad Cardenal Herrera-CEU, CEU Universities, Valencia, Spain; 6 Department of Pharmacy, Faculty of Health Sciences, Universidad Cardenal Herrera-CEU, CEU Universities, Valencia, Spain; 7 Botanical Garden, University of Valencia, Valencia, Spain

**Keywords:** black-tobacco, blond-tobacco, health impact, smoking-related diseases, tobacco consumption

## Abstract

**Background:**

There is ample scientific evidence to support that tobacco consumption is associated with diseases such as chronic obstructive pulmonary disease (COPD), bronchitis, and cancer. However, few studies have compared the harm caused by different types of tobacco, such as black and blond tobacco (BlaT, BloT).

**Objective:**

This study aims to search for the potential links between type of tobacco, dosage, tobacco consumption time, and disease.

**Methods:**

A longitudinal retrospective case–control study (163:162) was conducted using clinical histories and telephone interviews. Disproportions (prevalence, OR, and crude OR), dose and years of smoking (ANOVA), and strata (tobacco type, dose, and time) (Reg. Log. Bin. Cox) were used.

**Results:**

A significant correlation was discovered between smoking, type of tobacco, and disease, particularly for COPD. The OR for BlaT/non-smokers was 64.62 and for BloT/non-smokers was 26.44. When stratified by tobacco type, the crude OR for BlaT was 8.38 for COPD, 7.82 for bronchitis, and 2.25 for cancer. Cox R analysis revealed a dose–response relationship between smoking and time, with BlaT having Cox R values of 88.41 for COPD and 3.95 for cancer.

**Conclusion:**

The main conclusion is that while all types of tobacco are harmful, BlaT is even more harmful than BloT.

## 1 Introduction

Despite the globally implemented measures and strategies designed to prevent tobacco use and dependence, the consumption of conventional cigarettes remains widespread worldwide, and new trends continue to emerge with significant implications for public health. Robust evidence is available regarding the health effects of conventional cigarettes, water pipes and e-cigarettes. For instance, smokeless tobacco products have been associated not only with endothelial dysfunction and biomarkers of atherosclerosis, but also with emerging alterations in the proteome, transcriptome, epigenome, microbiome and circadian rhythm (1). Additional evidence compares the health effects of these recent trends with those of conventional combustible cigarette smoking, and notably, non-combustible products such as Swedish snus consistently demonstrate lower health risks than conventional cigarettes (2–4).

Although the studies cited provide valuable insights into the health effects of different tobacco products, our investigation focuses exclusively on conventional combustible cigarettes. This decision reflects the fact that smokeless tobacco products and electronic nicotine delivery systems involve fundamentally different exposure pathways and toxicological mechanisms, which are not comparable to those underlying chronic obstructive pulmonary disease (COPD) and bronchitis associated with burned tobacco. In addition, our approach is justified for several reasons. First, conventional cigarettes remain the most widely used tobacco product globally (5–11). Second, extensive and longstanding evidence has established a robust association between cigarette smoking and a wide range of diseases, particularly respiratory conditions such as COPD (12–18) and bronchitis (12, 14, 18, 19), as well as multiple forms of cancer (12, 13, 20–23). Moreover, the risk and severity of these diseases increase with both the extent of tobacco exposure and the duration of smoking (13, 24–27).

Research on the health effects of cigarette smoking is well established, and substantial evidence is available in this regard. However, considerably less is known about whether the type of tobacco used in manufactured cigarettes, blond or black tobacco (BloT, BlaT), results in differential health effects. Strengthening the scientific evidence in this area is particularly important given the widespread global consumption of both types of cigarettes.

The distinct processing methods and botanical varieties of *Nicotiana tabacum* L. used to produce BloT and BlaT result in chemical differences in the final product, irrespective of whether cigarettes are filtered or unfiltered. The differential chemical composition of black (air-cured, fermented) and blond (flue-cured, unfermented) tobacco has been extensively documented since the 1980s. These chemical divergences may have important biological implications. Urinary mutagenicity and exposure to aromatic amines have been shown to be significantly higher among smokers of BlaT (28), and air-cured tobaccos also contain higher concentrations of TSNAs (29). Epidemiological evidence further indicates increased risks of oesophageal, laryngeal and bladder cancers among consumers of BlaT (23), findings that are coherent with the carcinogenic constituents identified in the FDA’s list of Harmful and Potentially Harmful Constituents (HPHC), which is publicly available on the FDA website (30). Vineis and Pirastu (31) also identified compositional differences in aromatic amines between BlaT and BloT: BlaT contains higher concentrations of N-nitrosamines and 2-naphthylamines, and smokers of BlaT exhibit elevated blood levels of 4-aminobiphenyls as well as increased urinary mutagenicity (28, 29).

There is evidence suggesting that the type of tobacco influences health outcomes, with several studies reporting clear associations between specific cancer types and the use of BloT versus BlaT (21, 23, 32, 33). However, research directly comparing the health effects of smoking BloT and BlaT, particularly in relation to non-cancer outcomes, remains scarce. In this context, Zamora et al. (34, 35) conducted a preliminary population-based study addressing this question, although its interpretation was limited by a sample lacking a formal design and insufficient statistical power.

Building on this observation, studies employing designs that minimise random variability and ensure a sufficiently homogeneous population with adequately controlled characteristics are even rarer. This gap is especially evident in relation to respiratory diseases such as bronchitis and COPD. Given that the respiratory system is the organ system most directly exposed to tobacco smoke, differential responses to variations in tobacco type are biologically plausible (34–36). Addressing this lack of evidence is therefore essential to advancing our understanding of how the type of tobacco used in manufactured cigarettes may influence the risk of both malignant and non-malignant smoking-related diseases, particularly the latter, for which evidence is especially sparse.

Although several studies have reported associations between the use of BloTversus BlaT and specific cancer types (21, 23, 32, 33), much less is known about whether these two tobacco types differ in their effects on non-cancer outcomes. Only a few preliminary population-based studies, such as those by Zamora et al. (34, 35), have explored this question, although their interpretation is subject to limitations described earlier. In addition, studies with designs capable of minimising random variability and adequately controlling for key participant characteristics are particularly scarce, especially in relation to respiratory diseases such as bronchitis and COPD, despite these being the organ systems most directly exposed to tobacco smoke. Against this background, our study seeks to contribute more robust evidence by applying an *a priori* sample-size calculation, predefined case and control selection in a hospital setting, and a detailed exposure classification based on the tobacco brand consumed and the participant’s predominant tobacco type across their smoking lifetime. These methodological elements were intended to reduce confounding, improve the precision of our estimates, and help address the current evidence gap regarding the potential differential health effects of BloT and BlaT.

## 2 Methods

This study examined the association between smoking and three prevalent diseases among smokers: chronic obstructive pulmonary disease (COPD), bronchitis, and cancer. A stratified statistical analysis was conducted to explore associations, considering tobacco type (BloT/BlaT), smoking duration (“smoking years”), and dose (“cigarettes per day”). The methodology included logistic regression and odds ratios (OR) to assess initial associations, followed by Cox regression (Cox R) to estimate disease risk over time, providing a comprehensive evaluation of smoking-related health outcomes.

### 2.1 Study design

A retrospective paired case–control study at Consorcio Hospital General Universitario (Valencia, Spain) used medical records and telephone interviews to assess smoking-related diseases. Ethical approval from two independent ethics committees and informed consent were obtained; tobacco use and patient demographics were systematically recorded. Cases were defined as patients with ICD-9 codes 491/492 (corresponding to ICD-10: J40–J44) (37, 38), matched by age and gender with controls diagnosed outside these categories. Cases were recruited from the Pneumology Unit, controls from Traumatology, Ophthalmology, Internal Medicine, and Obstetrics. Patients from Psychiatry, Infectious Diseases, Nephrology, and Urology were excluded to minimise confounding. This design ensured comparability while reducing bias from underlying clinical characteristics. Smoking was the primary risk factor, defined as consuming over two cigarettes daily or maintaining an uninterrupted smoking history exceeding one year; both considered behaviours associated with increased disease risk.

### 2.2 Sample size

Following established protocols for paired retrospective designs (39–41), the theoretical sample consisted of 152 cases and 152 controls. To account for potential response lapses (20%) and diagnostic misclassification (50%), the sample was expanded to 258 cases and 258 controls. After adjusting for losses, the final study population included 163 cases and 162 controls, exceeding the *a priori* calculated minimum and ensuring sufficient statistical power.

### 2.3 Variables

Sociodemographic variables included sex and age. Smoking-related variables comprised presence/absence of smoking, tobacco type, smoking years (time), cigarettes per day (dose) and preferred tobacco. Disease-related variables encompassed the presence or absence of COPD, bronchitis, and cancer.

The type of tobacco was identified on the basis of the cigarette brand reported by each participant during the interview, which allowed classification into BlaT or BloT according to commercially established formulations. This categorisation is straightforward in the Spanish context, as black-tobacco cigarettes predominantly contain air-cured and fermented tobacco, typically associated with higher tar and nicotine yields, whereas blond-tobacco products consist mainly of flue-cured, non-fermented tobacco, which generally results in lower machine-measured tar levels. In addition to classifying the type of tobacco, it is important to note that each cigarette brand has its own manufacturing characteristics, including differences in filter design, blend composition and paper properties. Such variability may influence the effects produced by both BloTand BlaT cigarettes and should therefore be taken into account when interpreting results, particularly when findings appear unexpected.

### 2.4 Data analysis

Statistical evidence was derived through data analysis controlling for selection bias and estimating error probability. Qualitative variables were assessed using the Chi-square test; quantitative variables via Student’s t-test and mean differences. Contrasts involved Tobacco vs. Pathologies, Time, and Doses. OR with 95% confidence intervals (CI) were calculated, followed by stratified OR values by Tobacco type. The ‘Tobacco type’ was recoded as ‘Preferred tobacco’, excluding 10 patients who smoked multiple types. Stratified analyses yielded crude OR for each disease and for Black Tobacco, Blond Tobacco, and Non-smokers. Cox R was computed per disease using probability ratios across strata. Regression aimed to identify independent risk factors. Time was treated as constant, with risk function as the dependent variable, strengthening the epidemiological interpretation of observed associations (42, 43).

### 2.5 Ethical considerations: ethics committee approval and informed consent

The study was approved by two ethics committees (CEIC–Regional Ministry of Health and CEIC–CHGUV). Verbal informed consent was obtained from participants prior to the telephone interview.

## 3 Results

### 3.1 Smoking characteristics in the study population: cases and controls

Table 1 summarises the sample characteristics (*n* = 325; 265 males, 60 females). The mean age was 59.69 years for cases and 62.43 for controls. Smoking was reported by 238 participants (158 cases, 80 controls); 87 had never smoked (5 cases, 82 controls). BlaT was most used by cases (57.05%), BloT by controls (38.65%). Cases had longer initial hospitalisation and higher smoking prevalence. Significant differences were found in smoking status, duration (years), and intensity (cigarettes/day), with cases showing the highest values.

**Table 1:** Characteristics of patients.

	Cases		Controls	
Total	Male	Female	Male	Female
325	133	30	132	30

Age	Mean	*N*	Mean	*N*	Sig*
	59.69	163	62.43	162	0.002

Background	Cases	Controls	Sig**
Smokers	158 (96.93%)	80 (49.38%)	0.001
Smoking years	35.69	14.92	0.001
Black tobacco	93 (57.05%)	28 (17.28%)	0.001
Blond tobacco	63 (38.65%)	44 (27.16%)	0.001
Cigarettes per day	29.08	21.09	0.000
First cigarrette	16.13	16.94	0.103
No smoking years	6.62	9.85	0.003
Hospitalization days	7.33	5.15	0.001

Distribution of basic variables in cases and controls. Significance tests: Student’s t*; Chi square**.

### 3.2 Step 1: Association between smoking and disease in relation to duration and intensity

**Figure 1:**
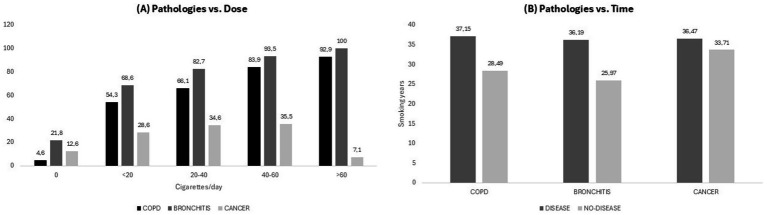
Relationship between three factors of smoking: pathologies, tobacco dose, and habit time (smoking years). **(A)** Pathologies vs. dose (*p* < 0.001); **(B)** pathologies vs. time [COPD and bronchitis: *p* < 0.001, cancer: *p* < 0.084; Dif. mean and 95% CI in COPD: 8.66 (5.6 to 11.70), bronchitis: 10.21 (6.36 to 14.06) and cancer: 2.75 (−0.37 to 5.89)]. Dose is cigarettes per day. Time is smoking years.

In the first part of the study, we examined the temporal and dose-dependent relationship between smoking and disease incidence. A positive correlation was observed between disease prevalence and both smoking dose and duration (Figure 1; Table 2). Smoking exposure was markedly higher among patients with COPD (49.8-fold), bronchitis (19.44-fold), and cancer (3.3-fold) (Table 3). Notably, 100% of individuals smoking over 60 cigarettes daily had bronchitis, and 92.9% had COPD, while only 7.1% of cancer patients reported this level of consumption, potentially due to survival bias. Over half of COPD and bronchitis cases occurred in individuals smoking fewer than 20 cigarettes daily. Across all conditions, affected individuals had significantly longer smoking histories than healthy counterparts. Mean smoking duration was highest in COPD (37.15 years), followed by cancer (36.68) and bronchitis (36.19). Disease development was associated with increased smoking years: bronchitis (10.21 years, 95% CI = 6.36–14.06, *p* = 0.001), COPD (8.66 years, 95% CI = 5.60–11.70, *p* = 0.001), and cancer (2.75 years, 95% CI = –0.37–5.89, *p* = 0.008).

**Table 2:** Means of smoking years according to pathologies.

	Smokers			
	Disease presence	No-disease	Mean difference and 95% CI	*p* <
COPD	37.15	28.49	8.66 (5.60–11.70)	0.001
Bronchitis	36.16	25.97	10.21 (6.36–14.06)	0.001
Cancer	36.47	33.71	2.75 (−0.37–5.89)	0.438

**Table 3:** Distribution of percentage of smoker and non-smokers according to pathologies.

	Smokers		No-smokers				
	Disease presence	No-disease	Disease presence	No-disease	OR	95% CI	Sig.
COPD	70.59	29.41	4.60	95.40	49.8	17.57–141.08	0.001
Bronchitis	84.46	15.54	21.84	78.16	19.44	10.48–36.06	0.001
Cancer	32.36	67.64	12.64	87.36	3.3	1.66–6.57	0.001

### 3.3 Step 2: Association between smoking, type of tobacco and disease

In Step 2, the relationship between smoking, tobacco type, and disease was analysed. OR analysis revealed that smoking BlaT was associated with greater disparities in disease prevalence, both in absolute terms -comparing smokers of a given tobacco type to non-smokers- and in relative terms -comparing BlaT smokers to BloT smokers (Table 4). This indicates a strong correlation between BlaT consumption and the studied pathologies. For COPD, patients were 64.62 times more likely to smoke BlaT compared to non-smokers, and 26.44 times more likely to smoke BloT. Similar trends were observed for bronchitis and cancer, although with lower OR values. Across all diseases, BlaT use was proportionally more frequent than BloT, with COPD showing the highest relative difference: BlaT was 2.44 times more frequent than BloT. These findings suggest that the type of tobacco consumed plays a significant role in disease development, with BlaT posing a higher risk than BloT across respiratory and oncological conditions.

**Table 4:** Tobacco type vs. pathologies according to the three isolated strata of the variable and ratio of disproportions between preferred tobacco and pathologies after recoding from tobacco type to preferred tobacco.

Strata	COPD			Bronchitis			Cancer		
	OR	95% CI	Sig.	OR	95% CI	Sig.	OR	95% CI	Sig.
Black tobacco vs. No smoking	64.62	25.17–165.88	0.001	20.87	10.20–42.69	0.001	3.38	1.72–6.66	0.001
Blond tobacco vs. No smoking	26.44	10.59–66.03	0.001	11.87	6.12–23.02	0.001	2.31	1.14–4.68	0.018
Black tobacco vs. Blond tobacco	2.44	1.34–4.46	0.003	1.75	0.83–3.68	0.132	1.46	0.83–2.57	0.180
Recoding tobacco type to preferred tobacco	Crude OR	95% CI	Sig.	Crude OR	95% CI	Sig.	Crude OR	95% CI	Sig.
Stratified	8.38	5.85–11.99	0.001	7.82	5.32–11.50	0.001	2.25	1.54–3.27	0.001

### 3.4 Step 3: Association between smoking, preferred tobacco and disease, according to time and dose

In a successive approach to causality, a stratified analysis was conducted on individuals who smoked exclusively one type of tobacco, allowing for the calculation of crude OR for the studied pathologies. As shown in Table 4, a history of smoking BlaT was 8.38 times more prevalent among COPD patients compared to non-smokers and BloT smokers. The crude OR of 8.38 and 7.82 indicate a strong association, as defined by Domínguez-Lara (44), with both values falling within 95% confidence intervals whose lower bounds exceed 5. These findings reflect disparities in absolute terms (smoking BlaT vs. not smoking) and relative terms (BlaT vs. BloT), suggesting that BlaT is more harmful than BloT and significantly more so than abstaining from tobacco.

Further analysis considered preferred tobacco in relation to dose and duration, as presented in Tables 5, 6. A clear dose-dependent correlation was observed: the proportion of affected individuals increased with the number of cigarettes smoked per day (Table 5). The highest incidence of disease occurred among individuals smoking 20–40 cigarettes daily, particularly among BlaT users. Within this group, 21.05% of COPD patients and 23.68% of bronchitis patients smoked BlaT. In contrast, among cancer patients smoking the same dose, BloT was the most frequently used tobacco. Regarding smoking duration, the highest proportion of COPD (25%) and bronchitis (27%) cases among BlaT smokers fell within the 20-40-year range (Table 5). Blond tobacco showed the strongest association with cancer. Notably, no cases of COPD or bronchitis were found among BloT smokers with over 40 years of smoking history, possibly due to survival bias, similar to that observed in high-dose groups.

**Table 5:** Distribution of percentages of patients with disease presence according to dose, time and preferred tobacco.

	COPD				Bronchitis				Cancer			
	BlaT		BloT		BlaT		BloT		BlaT		BloT	
	COPD	No-COPD	COPD	No-COPD	Bronchitis	No-bronchitis	Bronchitis	No-bronchitis	Cancer	No-cancer	Cancer	No-cancer
Cigarettes per day												
<20	3.51	4.82	0.44	5.26	3.95	6.14	0	3.95	1.75	2.63	2.19	7.46
20–40	21.05	15.35	7.46	10.09	23.68	20.61	4.82	4.82	12.28	5.70	16.23	19.74
40–60	16.23	6.14	2.19	1.32	17.10	7.46	1.32	0	5.26	4.39	13.16	3.07
>60	2.19	3.51	0	0.44	2.19	3.95	0	0	0	0.44	2.19	3.51
*p*	*p* < 0.038		*p* < 0.044		*p* < 0.024		*p* < 0.004		*p* < 0.205		*p* < 0.001	
Smoking years												
< 20	3.51	4.82	2.19	7.02	3.95	7.46	1.75	4.39	1.75	2.63	3.95	9.21
20–40	25.00	18.42	6.14	10.09	27.63	24.12	3.51	4.39	11.40	8.33	19.74	20.18
> 40	14.47	6.58	1.75	0	15.35	6.58	0.88	0	6.14	2.19	10.09	4.39
*p*	*p* < 0.001		*p* < 0.015		*p* < 0.011		*p* < 0.146		*p* < 0.009		*p* < 0.007	

Dose is cigarettes per day. Time is smoking years. Preferred tobacco is black tobacco (BlaT) or blon tobacco (BloT).

**Table 6:** Distribution of disease/non-disease ratio according to preferred tobacco, dose and time.

	COPD		Bronchitis		Cancer	
	Blast	BloT	BlaT	BloT	BlaT	BloT
	*r*	*r*	*r*	*r*	*r*	*r*
Cigarettes per day						
<20	0.73	0.08	0.64	0	0.67	0.3
20–40	1.37	0.74	1.15	1	2.15	0.82
40–60	2.64	1.66	2.29	*	1.2	4.3
>60	0.62	0	0.55	*	0	0.62
Smoking years						
< 20	0.73	0.31	0.53	0.4	0.67	0.43
20–40	1.36	0.61	1.15	0.8	1.37	0.98
> 40	2.66	*	2.33	*	2.8	2.3

Dose is cigarettes per day. Time is smoking years. Preferred tobacco is black tobacco (BlaT) or blon tobacco (BloT). r, disease presence/no-disease. (*), indefinite or indeterminate.

These patterns are further clarified by examining the ratio of sick to non-sick individuals (Table 6). This ratio increased significantly with doses above 20 cigarettes/day across all diseases, particularly at 20–40 cigarettes/day. For COPD and bronchitis, incidence was more than twice as high when BlaT was used (r = 2.64 for COPD; r = 2.29 for bronchitis). In contrast, cancer incidence was 4.3 times higher among BloT smokers at the same dose. At doses exceeding 60 cigarettes/day, results became paradoxical or indeterminate, potentially due to survival bias or economic constraints limiting consumption, especially of BloT.

### 3.5 Step 4: Association between the risk of disease according to preferred tobacco, dose, and time

Cox R (Table 7) was used to assess disease risk based on the tobacco type, dose, and smoking duration. Smoking BlaT significantly impacted COPD. Notably, all individuals smoking over 60 cigarettes/day had bronchitis, indicating an irrational probability. Compared to non-smokers, individuals who smoked BlaT were 88 times more likely to suffer from COPD, and 37 times more likely if they smoked BloT. BlaT use also raised bronchitis risk by 27.35 times. The risk of suffering from cancer is also significantly higher for BlaT smokers, with a 3.95-fold increase in likelihood. In summary, disease risk increased with higher doses and/or longer smoking duration, especially for COPD. BlaT was consistently associated with elevated risk across all pathologies studied.

**Table 7:** Probabilities’ ratio of disease vs. preferred tobacco, dose and time.

Preferred tobacco	COPD	Bronchitis	Cancer
Non-smokers	1	1	1
Black tobacco	88.41(29.39–265.95)	27.35(12.86–58.15)	3.94(1.89–8.21)
Blond tobacco	36.18(12.31–106.30)	15.56(7.70–31.46)	2.69(1.25–5.75)
Cigarettes per day			
Non-smoker	1	1	1
<20	24.64(7.39–82.11)	7.80(3.25–18.75)	2.76(1.04–7.27)
20–40	40.53(13.92–117.99)	17.08(8.60–33.90)	3.66(1.76–7.60)
40–60	107.90(32.16–361.92)	51.89(16.70–161.24)	3.80(1.67–8.61)
>60	269,75(27.92–2605.67)	*	0.53(0.06–4.47)
Smoking years			
Non smoker	1	1	1
< 20	16.42(5.09–52.90)	5.47(2.47–12.12)	2.09(0.81–5.40)
20–40	53.73(18.4–156.53)	24.25(11.9–49.3)	3.38(1.63–6.97)
> 40	142.28(39.60–511.16)	94.84(21.14–425.32)	4.26(1.85–9.82)

Cox regression. Variable 1st step: Time = 0. Dependent: Disease = 0/1. The reference category is: no-disease. (*) Overestimation beyond the whole range (all patients presented the disease [bronchitis]). Dose is cigarettes per day. Time is smoking years. Preferred tobacco is black tobacco or blond tobacco.

## 4 Discussion

As noted in the introduction, tobacco smoking has been linked to diseases such as COPD, bronchitis, and cancer. Prior studies have associated disease development with tobacco dose and smoking duration (13, 25, 27, 44, 45). These findings suggest a positive correlation between smoking intensity and disease prevalence, consistent with our results (Figure 1; Table 2). Increased tobacco dose or duration raised prevalence across all three pathologies. For cancer, high doses (>60 cigarettes/day) showed reduced prevalence, possibly due to survival bias and increased mortality.

The most compelling results concern tobacco type (BlaT vs. BloT) and disease incidence (Table 3), with BlaT strongly associated with COPD and bronchitis. Recoding tobacco type revealed a robust dose-time-disease relationship for BlaT (Tables 5, 6). Cox R (Table 7) confirmed BlaT’s greater harm, especially for COPD. It is true that successive analyses isolating the risk factor lead to a sustained decrease in sample size. Despite reduced sample size, OR remained high (2.44; 1.75; 1.46). The consistency and reproducibility of these associations are in line with the considerations outlined by Hill (41). Our findings suggest that the use of BlaT may be associated with a higher risk of the diseases examined compared with BloT. The differential health impact of the specific type of tobacco smoked (black versus blond), with BlaT generally appearing more harmful, is consistent with previous investigations. However, this evidence is particularly strong for cancer, whereas for non-cancer diseases the type of tobacco smoked is rarely distinguished, or the available scientific evidence remains limited.

Regarding cancer, De Stefani et al. (23) examined the association between tobacco type and oesophageal cancer and concluded that BlaT has a substantially greater harmful potential than BloT. Nevertheless, the classification of tobacco used in their analyses is not entirely clear, as the study groups together different tobacco forms (cigarettes, hand-rolled cigarettes, pipes and cigars) with tobacco types (black and blond), which may introduce confounding factors. Lee (32) conducted a comprehensive review of multiple heterogeneous studies, extracting and combining data to evaluate the relationships between tobacco type, tobacco form, tobacco components and their effects on lung cancer. He concluded that BlaT appears to be more harmful than other types and forms of tobacco. However, the wide heterogeneity of the included studies and the broad range of causal variables considered make it difficult to clearly isolate the effect attributable solely to BlaT, or to distinguish the specific impact of BlaT versus BloT. Nevertheless, there is a clear trend for BlaT to emerge as the most harmful option among the different causal variables examined. Samanic et al. (46) examined the risk associated with smoking only BlaT compared with smoking only BloT and found that bladder cancer risk was 40% higher among BlaT smokers. Molina-Montes et al. (33) investigated the possible association between tobacco type (black versus blond, and black versus non-smoking) and pancreatic cancer. They reported that, compared with never-smokers, BlaT smokers exhibited a significantly higher risk of pancreatic cancer, and that this tobacco type appeared to be more harmful than BloT. Taken together, and despite the methodological differences across studies, the potential confounding factors involved, and the contrasts with our own study design, all findings consistently point towards a close relationship between smoking BlaT and an increased risk of cancer.

As highlighted in the introduction, very few studies have directly examined the differential impact of BloT versus BlaT on COPD or bronchitis. Among them, the study by Zamora et al. (35) stands out, they found higher disease incidence among BlaT smokers, attributing toxicity to its chemical and physical properties. These findings are broadly consistent with ours. However, the study presents several limitations, including a small sample size, a design susceptible to substantial random variation and confounding, and reliance on self-reported information without clinical verification. Despite these weaknesses, the evidence indicates that BlaT is a more significant risk factor for respiratory diseases such as COPD and bronchitis, which is consistent with our findings.

The higher risk observed in BlaT smokers is consistent with the compositional differences between BloT and BlaT, with the latter containing higher levels of carcinogens and respiratory toxicants, according to the evidence cited in the introduction. These chemical characteristics offer a plausible explanation for the greater incidence of bronchitis, COPD and cancer associated with smoking BlaT. However, tobacco composition is not the only factor that may influence disease risk, and our study did not explore other potential determinants such as smoking topography, combustion temperature, cigarette construction, additives or social and behavioural factors. Therefore, while the chemical profile of BlaT is compatible with the epidemiological patterns described, the contribution of additional unmeasured factors cannot be excluded. To minimise potential confounding as far as possible, our study design restricted both case and control recruitment to a single hospital setting. A preliminary sample size calculation was performed, and cases and controls were classified according to the criteria detailed in the Methods section. Furthermore, BloT and BlaT were clearly defined, and participants’ smoking habits were classified according to the composition of the cigarette brand they reported using.

Given that this study derives from research on tobacco, a field already supported by an extensive body of evidence, one might reasonably ask: “What new insights does this study offer?” To address this question, we highlight the study’s strengths, which enhance the robustness and relevance of its conclusions and distinguish it from some previous studies.

Firstly, its design seeks to minimise confounding factors through several key methodological decisions: the use of a homogeneous setting from which the study population is drawn; a clear definition of cases and controls; robust selection criteria for both groups; a precise delineation of the risk factor under investigation (conventional BlaT cigarettes versus conventional BloT cigarettes); the selection of specific diseases, particularly bronchitis and COPD; and a progressive analytical approach to causality. This is a controlled study that provides a detailed assessment of the relationship between tobacco type, dose, and duration, using multiple statistical approaches, including the Cox R model, to evaluate disease risk. Moreover, the study incorporates the variable “preferred tobacco type”, enabling a more accurate assessment of its impact on disease incidence, particularly in COPD and bronchitis, for which there is limited scientific evidence regarding the influence of tobacco type in conventional cigarettes. Secondly, the consistency of the results across the different analytical models and the reproducibility of the associations, even with relatively small sample sizes, support the reliability of the findings. Finally, the integration of clinically relevant epidemiological evidence strengthens the case for a review of tobacco control policies.

It is also important to note that, given the high prevalence and substantial societal burden of smoking-related diseases (47, 48), together with the scarcity of studies considering tobacco type in relation to non-cancer outcomes, further research into how specific smoking characteristics influence disease development is essential. Such knowledge could inform the design of more effective prevention strategies and smoking cessation interventions (49–54).

Although this study is of considerable interest given its public health implications, several limitations should be acknowledged, as they may potentially introduce residual confounding, despite the fact that the study design was originally intended to minimise such effects. In particular, cases and controls were recruited from different hospital units, which may imply differences in patient profiles. In addition, the analysis does not include a clearly defined and robust multivariable adjustment for key factors such as alcohol consumption, occupation, socioeconomic status, environmental exposure, cigarette characteristics or comorbidities.

Another limitation relates to the progressive reduction in sample size in the stratified analyses, which may reduce statistical power, although it does not undermine the consistency of the observed associations. Furthermore, the observational nature of the study inevitably limits causal inference, as unmeasured or insufficiently controlled factors may still be present despite the strength and coherence of the associations identified. As with all population-based studies, intrinsic inter-individual variability, together with external and internal factors related both to cigarette characteristics and to the type of tobacco used in their manufacture, may influence the results. These include features such as cigarette paper, the presence and type of filter, the degree of tobacco curing and the blend of botanical varieties. The scarcity of comparative studies focusing specifically on black tobacco (BlaT) and blond tobacco (BloT) further limits external validation. Finally, the reliance on self-reported smoking data introduces the possibility of recall bias.

Nevertheless, despite these acknowledged limitations, the methodological rigour of the study and the consistency of the findings across the different analytical approaches support the interpretation that differences in harm may exist between the two types of tobacco. This consideration may be of relevance for the clinical management of patients affected by COPD, bronchitis and cancer.

With regard to future research directions, studies should aim to address current limitations by employing larger and more diverse samples, while maintaining consistent inclusion criteria and study design, to enhance statistical power and comparability. Longitudinal or prospective cohort designs would be particularly valuable in clarifying causal relationships between tobacco type, dose and duration, and the onset of COPD and bronchitis, for which the present study provides the most novel evidence. Standardising the recording of “type of tobacco” as a routine variable in epidemiological studies, clinical research and patient medical histories would substantially improve precision and facilitate cross-study comparisons. In addition, incorporating objective measures of tobacco exposure, such as biomarkers or validated environmental assessments, could minimise recall bias and strengthen data reliability. Finally, future work should expand comparative analyses of BlaT and BloT, helping to validate and extend the findings presented here.

Taken together, these considerations highlight the need for more refined research designs, while reinforcing the contribution of the present study in providing robust evidence that the type of tobacco used has a meaningful impact on the risk of COPD and bronchitis. These findings form the basis for the conclusions presented below.

## 5 Conclusion

This study provides evidence that the type of tobacco used in conventional cigarettes is associated with differences in the risk of COPD and bronchitis, and it also suggests an increased cancer risk associated with black tobacco (BlaT). Across all conditions examined, BlaT was consistently associated with a higher incidence. The methodological strengths of the study, particularly population homogeneity (single-hospital setting) and the use of multiple analytical approaches, support the internal consistency of these findings. Nevertheless, in view of the study’s inherent limitations, it would be inappropriate to draw definitive conclusions that fully resolve this issue. Further research is therefore required, incorporating variables not addressed in the present study and study designs that reduce, and where possible eliminate, reliance on participants’ recollection of their own tobacco consumption. Addressing these challenges represents a demanding yet valuable direction for future research. Overall, even when the study limitations are taken into account, the consistency of the observed associations supports the interpretation of the findings.

These results underscore the importance of incorporating tobacco type into epidemiological analyses, clinical assessments and tobacco control policies. Public health strategies should consider the differential risks associated with BlaT and BloT, particularly in populations with high consumption of BlaT cigarettes.

## Data Availability

The raw data supporting the conclusions of this article will be made available by the authors, without undue reservation.

## References

[1] Münzel T Hahad O Kuntic M Keaney JF Deanfield JE Daiber A. Effects of tobacco cigarettes, e-cigarettes, and waterpipe smoking on endothelial function and clinical outcomes. Eur Heart J. (2020) 41:4057–70. doi: 10.1093/eurheartj/ehaa460 32585699 PMC7454514

[2] Lee PN. Epidemiological evidence relating snus to health--an updated review based on recent publications. Harm Reduct J. (2013) 10:36. doi: 10.1186/1477-7517-10-36 24314326 PMC4029226

[3] Lee PN Farsalinos K. Comparing smoking-related disease rates from e-cigarette use with those from tobacco cigarette use: a reanalysis of a recently-published study. Harm Reduct J. (2025) 22:78. doi: 10.1186/s12954-025-01230-y 40361147 PMC12070775

[4] Lee PN Coombs KJ Fry JS. Estimating lung cancer risk from e-cigarettes and heated tobacco products: applications of a tool based on biomarkers of exposure and of potential harm. Harm Reduct J. (2025) 22:45. doi: 10.1186/s12954-025-01188-x 40159472 PMC11955122

[5] Institute for Health Metrics and Evaluation (IHME). Findings from the Global Burden of Disease Study. Seattle, WA: Institute for Health Metrics and Evaluation (2017). p. 2018.

[6] Kotz D Böckmann M Kastaun S. The use of tobacco, E-cigarettes, and methods to quit smoking in Germany. Dtsch Arztebl Int. (2018) 115:235–42. doi: 10.3238/arztebl.2018.0235 29716687 PMC5938545

[7] Schilling L Spallek J Maul H Tallarek M Schneider S. Active and passive exposure to tobacco and e-cigarettes during pregnancy. Matern Child Health J. (2021) 25:656–65. doi: 10.1007/s10995-020-03037-8 33211261 PMC8032614

[8] World Health Organization. Global action plan for the Prevention and Control of Noncommunicable Diseases 2013–2020. Geneva: World Health Organization (2013).

[9] World Health Organization. WHO Global Report on Trends in Prevalence of Tobacco Smoking 2015. Geneva: World Health Organization (2015).

[10] World Health Organization. WHO Global Report on Trends in Prevalence of Tobacco Smoking 2000–2025. 2nd ed. Geneva: World Health Organization (2018).

[11] World Health Organization. WHO Global Report on Trends in Prevalence of Tobacco Smoking, 2000–2025. 3rd ed. Geneva: World Health Organization (2019).

[12] Chan KH Wright N Xiao D Guo Y Chen Y Du H . Tobacco smoking and risks of more than 470 diseases in China: a prospective cohort study. Lancet Public Health. (2022) 7:e1014–26. doi: 10.1016/S2468-2667(22)00227-4 36462513 PMC7613927

[13] Dai X Gil GF Reitsma MB Ahmad NS Anderson JA Bisignano C . Health effects associated with smoking: a burden of proof study. Nat Med. (2022) 28:2045–55. doi: 10.1038/s41591-022-01978-x 36216941 PMC9556318

[14] Goren A Gupta S Dong P Feng Y Chen C Liu D. Burden of smoking among adults with COPD, chronic bronchitis, and emphysema in urban China. Int J Clin Pract. (2015) 69:1015–28. doi: 10.1111/ijcp.12680 26136208

[15] Müllerova H Maselli DJ Locantore N Vestbo J Hurst JR Wedzicha JA . Hospitalized exacerbations of COPD: risk factors and outcomes in the ECLIPSE cohort. Chest. (2015) 147:999–1007. doi: 10.1378/chest.14-0655 25356881

[16] Maremanda KP Sundar IK Rahman I. Role of inner mitochondrial protein OPA1 in mitochondrial dysfunction by tobacco smoking and in the pathogenesis of COPD. Redox Biol. (2021) 45:102055. doi: 10.1016/j.redox.2021.102055 34214709 PMC8258692

[17] Ni Y Shi G Qu J. Indoor PM2.5, tobacco smoking and chronic lung diseases: a narrative review. Environ Res. (2020) 181:108910. doi: 10.1016/j.envres.2019.108910 31780052

[18] Paulose-Ram R Tilert T Dillon CF Brody DJ. Cigarette smoking and lung obstruction among adults aged 40-79: United States, 2007-2012. NCHS Data Brief. (2015) 81:1–8. 25569298

[19] Pahwa P Karunanayake CP Rennie DC Lawson JA Ramsden VR McMullin K . Prevalence and associated risk factors of chronic bronchitis in first nations people. BMC Pulm Med. (2017) 17:95. doi: 10.1186/s12890-017-0432-4, 28662706 PMC5492442

[20] Working IARC. Group on the evaluation of carcinogenic risks to humans. Tobacco smoke and involuntary smoking. IARC Monogr Eval Carcinog Risks Hum. (2004) 83:1–1438. 15285078 PMC4781536

[21] Jung KJ Jeon C Jee SH. The effect of smoking on lung cancer: ethnic differences and the smoking paradox. Epidemiol Health. (2016) 38:e2016060. doi: 10.4178/epih.e2016060 28092929 PMC5309724

[22] Peleteiro B Castro C Morais S Ferro A Lunet N. Worldwide burden of gastric cancer attributable to tobacco smoking in 2012 and predictions for 2020. Dig Dis Sci. (2015) 60:2470–6. doi: 10.1007/s10620-015-3624-x, 25786860

[23] De Stefani E Barrios E Fierro L. Black (air-cured) and blond (flue-cured) tobacco and cancer risk. III: oesophageal cancer. Eur J Cancer. (1993) 29A:763–6. doi: 10.1016/s0959-8049(05)80363-6 8471336

[24] Inoue-Choi M Christensen CH Rostron BL Cosgrove CM Reyes-Guzman C Apelberg B . Dose-response Association of low-Intensity and Nondaily Smoking with Mortality in the United States. JAMA Netw Open. (2020) 3:e206436. doi: 10.1001/jamanetworkopen.2020.6436 32492162 PMC7272118

[25] Lévêque E Lacourt A Philipps V Luce D Guénel P Stücker I . A new trajectory approach for investigating the association between an environmental or occupational exposure over lifetime and the risk of chronic disease: application to smoking, asbestos, and lung cancer. PLoS One. (2020) 15:e0236736. doi: 10.1371/journal.pone.0236736 32785269 PMC7423115

[26] Luo J Tang X Li F Wen H Wang L Ge S . Cigarette smoking and risk of different pathologic types of stroke: a systematic review and dose-response meta-analysis. Front Neurol. (2022) 12:772373. doi: 10.3389/fneur.2021.772373 35145466 PMC8821532

[27] Zhang S Chen H Wang A Liu Y Hou H Hu Q. Assessment of genotoxicity of four volatile pollutants from cigarette smoke based on the in vitro γH2AX assay using high content screening. Environ Toxicol Pharmacol. (2017) 55:30–6. doi: 10.1016/j.etap.2017.07.005 28818740

[28] Malaveille C Vineis P Estéve J Ohshima H Brun G Hautefeuille A . Levels of mutagens in the urine of smokers of black and blond tobacco correlate with their risk of bladder cancer. Carcinogenesis. (1989) 10:577–86. doi: 10.1093/carcin/10.3.577 2924402

[29] Ding YS Zhang L Jain RB Jain N Wang RY Ashley DL . Levels of tobacco-specific nitrosamines and polycyclic aromatic hydrocarbons in mainstream smoke from different tobacco varieties. Cancer Epidemiol Biomarkers Prev. (2008) 17:3366–71. doi: 10.1158/1055-9965.EPI-08-0320 19064552

[30] Food and Drug Administration (FDA). Harmful and Potentially Harmful Constituents in Tobacco Products and Tobacco Smoke: Established List. U.S. Department of Health and Human Services. Available online at: https://www.fda.gov/tobacco-products/rules-regulations-and-guidance-related-tobacco-products/harmful-and-potentially-harmful-constituents-tobacco-products-and-tobacco-smoke-established-list (Accessed April 1, 2026).

[31] Vineis P Pirastu R. Aromatic amines and cancer. Cancer Causes Control. (1997) 8:346–55. doi: 10.1023/a:1018453104303 9498898

[32] Lee PN. Lung cancer and type of cigarette smoked. Inhal Toxicol. (2001) 13:951–76. doi: 10.1080/089583701753210353 11696868

[33] Molina-Montes E Van Hoogstraten L Gomez-Rubio P Löhr M Sharp L Molero X . Pancreatic cancer risk in relation to lifetime smoking patterns, tobacco type, and dose-response relationships. Cancer Epidemiol Biomarkers Prev. (2020) 29:1009–18. doi: 10.1158/1055-9965.EPI-19-1027 32051190

[34] Zamora MJ Bejarano-Vila M Bosch F Martínez-Solís I Hermenegildo C Alonso MJ. Influencia del tabaco en la salud (I): Contaminaciones en el tabaco. Farmacéutico (Barc). (2009) 418:38–44.

[35] Zamora MJ Bejarano-Vila M Bosch F Martínez-Solís I Hermenegildo C Alonso MJ. Influencia del tipo de tabaco en la salud (II): efectos del tabaquismo. Farmacéutico (Barc.). (2009) 419:60–8.

[36] Borgerding MF Bodnar JA Curtin GM Swauger JE. The chemical composition of smokeless tobacco: a survey of products sold in the United States in 2006 and 2007. Regul Toxicol Pharmacol. (2012) 64:367–87. doi: 10.1016/j.yrtph.2012.09.003 23000415

[37] National Center for Health Statistics (U.S.), Council on Clinical Classifications, Commission on Professional and Hospital Activities, & World Health Organization. International Classification of Diseases, 9th Revision (ICD‑9). Geneva: World Health Organization; (1978).

[38] World Health Organization. ICD-10: International Statistical Classification of Diseases and Related Health Problems: tenth Revision, 2nd ed. World Health Organization. (2004). Available online at: https://iris.who.int/handle/10665/42980 (Accessed April 1, 2026).

[39] Pértegas Díaz S Pita-Fernández S. Cálculo del tamaño muestral en estudios de casos y controles. Cad Aten Primaria. (2002) 9:148–50.

[40] Fletcher RH Fletcher SW Wagner EH. Clinical Epidemiology: The Essentials. 3rd ed. Baltimore: Williams and Wilkins (1996).

[41] Hill AB. Environmental and disease: association or causation? Proc R Soc Med. (1965) 58:295–300. doi: 10.1177/003591576505800503, 14283879 PMC1898525

[42] Lucas RM., McMichael AJ. Association or causation: evaluating links between "environment and disease". Bull World Health Organ (2005) 83: 792–795. Available online at: https://apps.who.int/iris/handle/10665/269505, . 16283057 PMC2626424

[43] Domínguez-Lara SA. El odds ratio y su interpretación como magnitud del efecto en investigación. Rev Esp Educ Méd. (2018) 19:65–6. doi: 10.1016/j.edumed.2017.01.008

[44] AdeD D Gimeno JC Ferrer MJ Fabregas ML Folch PA Paya JM. A study of the effect of proinflammatory cytokines on the epithelial cells of smokers, with or without COPD. Arch Bronconeumol. (2011) 47:447–53. doi: 10.1016/j.arbres.2011.04.007 21676518

[45] Rasmussen JE Sheridan JT Polk W Davies CM Tarran R. Cigarette smoke-induced Ca2+ release leads to cystic fibrosis transmembrane conductance regulator (CFTR) dysfunction. J Biol Chem. (2014) 289:7671–81. doi: 10.1074/jbc.M113.545137 24448802 PMC3953278

[46] Samanic C Kogevinas M Dosemeci M Malats N Real FX Garcia-Closas M . Smoking and bladder cancer in Spain: effects of tobacco type, timing, environmental tobacco smoke, and gender. Cancer Epidemiol Biomarkers Prev. (2006) 15:1348–54. doi: 10.1158/1055-9965.EPI-06-0021 16835335

[47] Chapel JM Ritchey MD Zhang D Wang G. Prevalence and medical costs of chronic diseases among adult Medicaid beneficiaries. Am J Prev Med. (2017) 53:S143–54. doi: 10.1016/j.amepre.2017.07.019 29153115 PMC5798200

[48] Jethwa AR Khariwala SS. Tobacco-related carcinogenesis in head and neck cancer. Cancer Metastasis Rev. (2017) 36:411–23. doi: 10.1007/s10555-017-9689-6 28801840 PMC5709040

[49] Abu Ghazaleh H Mulnier H Duaso M. A qualitative approach exploring the experiences of smoking and quitting attempts in type 1 diabetes. J Clin Nurs. (2018) 27:3091–103. doi: 10.1111/jocn.14499 29700882

[50] Albedah AM Khalil MK Khalil AA Elolemy AT. Use of the target group index survey to evaluate the cigarette smoking profile in Saudi Arabia. Saudi Med J. (2011) 32:1055–9. doi: 10.15537/1658-3175.5400, 22008927

[51] Becoña E Vázquez MI MdelC M Fernández del Río E López-Durán A Martínez Ú . Smoking habit profile and health-related quality of life. Psicothema. (2013) 25:421–6. doi: 10.7334/psicothema2013.73 24124772

[52] Buchanan T Magee CA Igwe EO Kelly PJ. Is the Australian smoking population hardening? Addict Behav. (2021) 112:106575. doi: 10.1016/j.addbeh.2020.106575 32871404

[53] Jackson SE Shahab L Garnett C Brown J. Trends in and correlates of use of roll-your-own cigarettes: a population study in England 2008-2017. Nicotine Tob Res. (2020) 22:942–9. doi: 10.1093/ntr/ntz082 31095329

[54] Patterson F Grandner MA Lozano A Satti A Ma G. Transitioning from adequate to inadequate sleep duration associated with higher smoking rate and greater nicotine dependence in a population sample. Addict Behav. (2018) 77:47–50. doi: 10.1016/j.addbeh.2017.09.011 28950118 PMC5701829

